# Development of an eHealth Mindfulness-Based Music Therapy Intervention for Adults Undergoing Allogeneic Hematopoietic Stem Cell Transplantation: Qualitative Study

**DOI:** 10.2196/65188

**Published:** 2025-04-11

**Authors:** Sara E Fleszar-Pavlovic, Blanca Noriega Esquives, Padideh Lovan, Arianna E Brito, Ann Marie Sia, Mary Adelyn Kauffman, Maria Lopes, Patricia I Moreno, Tulay Koru-Sengul, Rui Gong, Trent Wang, Eric D Wieder, Maria Rueda-Lara, Michael Antoni, Krishna Komanduri, Teresa Lesiuk, Frank J Penedo

**Affiliations:** 1 Sylvester Comprehensive Cancer Center, University of Miami Miller School of Medicine, Miami, FL, United States; 2 School of Nursing and Health Studies, University of Miami Miller School of Medicine, Miami, FL, United States; 3 Department of Undergraduate Research, University of Miami, Coral Gables, FL, United States; 4 Department of Psychology, College of Arts and Sciences, University of Miami, Coral Gables, FL, United States; 5 Department of Public Health Sciences, University of Miami Miller School of Medicine, Miami, FL, United States; 6 Department of Medicine, University of Miami Miller School of Medicine, Miami, FL, United States; 7 Department of Psychiatry and Behavioral Sciences, University of Miami Miller School of Medicine, Miami, FL, United States; 8 Departments of Psychology and Medicine, University of Miami Miller School of Medicine, 1120 NW 14th Street, Miami, FL, 33136-1002, United States; 9 Department of Medicine, Helen Diller Family Comprehensive Cancer Center, University of California, San Francisco, San Francisco, CA, United States; 10 Frost School of Music, University of Miami, Coral Gables, FL, United States

**Keywords:** allogeneic stem cell transplantation, hematologic malignancy, bone marrow transplant, mindfulness-based music therapy, mindfulness, music therapy, eHealth, music therapy intervention, adult, adolescence, allogeneic, stem cell transplantation, stem cell, transplantation, qualitative study, treatment, hematologic cancers, psychological distress, side effects, mindfulness-based stress reduction, stress reduction, anxiety, depression, diagnosis, blood sample collection, eHealth tool, quality of life, cancer survivors

## Abstract

**Background:**

Allogeneic hematopoietic stem cell transplantation (allo-SCT) is an effective treatment for various hematologic cancers, though it often results in severe side effects and psychological distress, which can negatively impact health outcomes. Integrative therapies like mindfulness-based stress reduction (MBSR), mindfulness meditation (MM), and music therapy (MT) yield promising results in enhancing both psychosocial outcomes (eg, reducing anxiety and depression) and physiological adaptation (eg, decreasing inflammation) in cancer patients.

**Objective:**

We developed and refined, using focus groups and environmental and field testing, an eHealth-delivered mindfulness-based music therapy (eMBMT) intervention aimed at improving health-related quality of life, symptom burden (ie, pain, fatigue, and sleep), disease activity (ie, chronic graft-versus-host disease, cytomegalovirus activation, and infections) and psychosocial (ie, depression, anxiety, and cancer-specific distress) and physiological adaptation (ie, inflammation and immune reconstitution) tailored to adults receiving allo-SCT.

**Methods:**

eMBMT intervention content is grounded in MT, MM, and MBSR, developed by a multidisciplinary team, and adapted for adults undergoing allo-SCT. eMBMT content was refined through focus groups and usability and field testing. Focus groups used a semistructured interview guide, while field testing used the “think aloud” method. Usability was evaluated using the 30-item Usefulness, Satisfaction, and Ease of Use (USE) questionnaire. Descriptive statistics analyzed the USE questionnaire and participant characteristics, while rapid qualitative analysis was applied to focus groups and field-testing sessions. Survivors eligible to participate in the focus groups and usability and field testing were adults (>18 years old) who received an allo-SCT (<36 months) for myelodysplastic syndrome, acute myeloid leukemia, or chronic myeloid leukemia, and were in remission for greater than 3 months.

**Results:**

During the focus groups, participants (n=11; mean age 43.6, SD 17.8 years) provided qualitative feedback highlighting the shock of diagnosis, challenges during hospitalization, and coping strategies posttreatment. The eMBMT platform received positive evaluations for usefulness (mean 6.47, SD 0.29), ease of use (mean 6.92, SD 0.60), and satisfaction (mean 6.16, SD 0.82). Key themes from field testing highlighted the significance of social support, hope, and maintaining an active lifestyle. Suggestions for improvement included incorporating more representative content, reducing text, enhancing guidance, offering diverse music options, and streamlining blood sample collection.

**Conclusions:**

The eMBMT intervention is a comprehensive, user-friendly eHealth tool tailored to the unique needs of allo-SCT patients. The positive feedback and identified areas for improvement underscore its potential to enhance well-being, symptom management, and overall quality of life for cancer survivors. A future pilot randomized controlled trial will further evaluate the feasibility, acceptability, and preliminary efficacy of the eMBMT intervention in improving health-related quality of life, symptom burden, disease activity, and psychosocial and physiological adaptation.

## Introduction

In the United States, hematologic malignancies account for approximately 10% of all new cancer diagnoses and 9.4% of cancer-related deaths annually [1]. It is projected that over 180,000 new cases of malignant hematologic cancers will be diagnosed in 2024 [2]. Allogeneic hematopoietic stem cell transplantation (allo-SCT) is an effective therapy for various hematologic cancers, including acute myeloid leukemia, chronic myeloid leukemia, and myelodysplastic syndrome [3]. Allo-SCT is a multiphase therapy starting with a pretransplant evaluation and finding a compatible donor. This is followed by intensive conditioning (chemotherapy, radiation, or both) to destroy cancer cells, suppress the immune system, and prepare for stem cell engraftment. The patient then receives the donor’s stem cells intravenously and enters the posttransplant phase, focusing on engraftment and monitoring for complications like graft-versus-host disease (GVHD) and infections [3].

Despite its curative potential, allo-SCT may be accompanied by negative side effects (eg, nausea, vomiting, pain, fatigue, mood disturbance, and risk of infections) [4]. In addition, poor psychosocial functioning and emotional distress (eg, anxiety, depression, and social isolation) can persist well beyond active treatment [5-7]. These negative psychological factors, common among allo-SCT recipients [5,6], are associated with higher mortality rates and less favorable health outcomes in the first-year posttreatment [8-10], including greater rates of GVHD and infection, longer hospitalization stays, and higher readmission rates [11]. Furthermore, research indicates that psychological distress can affect physiological responses to treatment such as increased inflammation [12,13]. For example, in patients receiving allo-SCT, pretransplant anxiety, depression, and stress have been associated with delayed white blood cell recovery, slower immune function recovery, and shorter survival. In contrast, pretransplant optimism, positive affect, and social support correlate with a shorter time to engraftment and reduced incidence of GVHD [14-17]. Therefore, interventions targeting psychological distress and physical burden in allo-SCT recipients may enhance health-related quality of life (HRQoL), improve patient-reported outcomes, and support immune recovery and overall health in cancer survivors.

A growing body of research suggests that mindfulness-based interventions, including mindfulness-based stress reduction (MBSR) and mindfulness meditation (MM) yield promising results in enhancing both psychosocial outcomes (eg, reducing anxiety and depression) and physiological adaptation (eg, decreasing inflammation) in cancer patients [18-21]. The core principle of mindfulness involves maintaining nonjudgmental awareness of the present moment, which includes recognizing and accepting one’s thoughts, emotions, and bodily sensations as they arise. Practicing mindfulness fosters a greater sense of self-awareness and emotional regulation, which can contribute to improved mental and physical well-being [22]. Although highly underused, mindfulness-based interventions have proven effective in alleviating cancer symptoms, cancer-related distress, and depression in various patient populations. Mindfulness-based interventions also enhance self-efficacy, coping skills, and HRQoL in cancer patients [12,20,23-27] and individuals with other chronic diseases [28-31].

In addition to mindfulness, music therapy (MT) is a well-recognized approach for addressing various psychosocial and physiological needs in clinical populations [32-44]. It has demonstrated effectiveness in reducing stress and enhancing emotional well-being among cancer patients [35,45-49]. MT offers numerous benefits for cancer patients by using both interactive (eg, singing) and receptive techniques (eg, listening to music and music imagery) to improve mood, reduce stress, pain, and anxiety, as well as promote relaxation [50]. Although most findings suggest psychological improvement associated with MT, a recent meta-analysis of controlled trials provided evidence that MT can significantly improve physical symptoms (eg, fatigue) and reduce pain in cancer patients [51,52]. MT can also serve as a foundation for planning effective rehabilitation programs and improving overall quality of life [50]. Using MT is known to be a low-risk, minimally invasive, and cost-effective approach to managing patients’ well-being particularly in the often-stressful environment of a cancer clinic [53].

Although previous research has demonstrated that practices related to mindfulness and MT can positively affect fatigue, pain, anxiety, and HRQoL in cancer patients [54,55], there is a lack of well-powered randomized controlled trials and reproducible interventions specific for allo-SCT patients. In addition, significant gaps persist in our understanding of how specific components of mindfulness and MT impact psychosocial and physiological adaptation, HRQoL, and overall health outcomes in this population. Therefore, we designed an eHealth-delivered mindfulness-based music therapy (eMBMT) aimed at reducing symptom burden and improving HRQoL in patients undergoing allo-SCT. We selected an eHealth intervention based on its proven advantages, including patient accessibility and convenience, improved communication, and reduced burden on health care systems. Overall eHealth interventions offer a valuable approach to complement traditional cancer care [56]. This study aims to refine and finalize, using focus groups and environmental and field testing, the newly developed eMBMT intervention.

## Methods

### Intervention Content Development

The eMBMT content was developed by a multidisciplinary team (eg, board-certified music therapists [MT-BC], psychologists, oncologists, and psychiatrists) and is based on MT, MM, MBSR [57], and Lesiuk’s [58,59] MBMT pilot trial with breast cancer survivors. eMBMT was further adapted for patients undergoing allo-SCT and structured to be delivered through BrightOutcome’s SmartManage: Tools for Health Living [60,61] platform. SmartManage is a patient-centered web application that is easy to use, Health Insurance Portability and Accountability Act (HIPAA) compliant, and accessible through multiple platforms (ie, tablets and smartphones). SmartManage serves as the primary platform for hosting the MBMT content, organized into 8 modules corresponding to sessions led by the MT-BC. SmartManage enables participants to engage in real time during sessions and supports independent practice by providing access to session materials, guided exercises, and additional resources for extended learning. Participants were encouraged to engage with SmartManage outside of scheduled sessions, using its resources to review session materials and independently practice intervention components at their own pace, thereby reinforcing the therapeutic process. The eMBMT program consists of eight 60-minute sessions led by a MT-BC. The sessions integrate MT and mindfulness attitudes (ie, nonjudging, patience, letting go, acceptance, trust, beginners mind, and nonstriving) developed by Kabat-Zinn [57]. MT-BCs guide patients in engaging with video and audio materials, practicing active and receptive music therapy exercises, incorporating music and journaling, and participating in video conferencing, all aimed at fostering mindfulness attitudes through music therapy. eMBMT’s 8 tailored sessions span the allo-SCT treatment and recovery journey beginning upon hospital admission (approximately 7 days before transplant) and ending postdischarge (approximately 75‐115 days posttransplant).

eMBMT begins with an orientation session to provide an overview of the program and its aims. The following 7 sessions are aligned with the patient’s treatment trajectory, each emphasizing a specific mindfulness attitude to be practiced throughout their allo-SCT treatment: (1) nonjudging encourages evaluating stimuli without habitual reactions, whether from internal dialogue, sensations, or external events; (2) patience helps manage worry by allowing events to unfold over time; (3) letting go involves accepting things as they are and being open to change; (4) acceptance focuses on viewing one’s experience without comparisons and allowing recovery to unfold at its own pace; (5) trust emphasizes believing in oneself and empowering self-reliance over thoughts; (6) beginner’s mind encourages seeing newness in familiar environments, fostering a sense of wonder, and shedding negative perceptions; and (7) nonstriving promotes the willingness to try new experiences and suspend critical judgments, particularly through new music experiences. MT-BCs focus on guiding patients through each session to first learn the attitude through music exercises, then how to apply the attitude to potentially improve their treatment journey.

### Intervention Refinement

Following the initial content development, we used focus groups and usability and field-testing sessions to refine and finalize the eMBMT content and intervention.

#### Sampling and Recruitment

Participants were recruited by (a) physician referral or (b) the University of Miami’s consent-to-contact database to participate in a focus group and a subsequent one-on-one usability and field-testing session. Patients at the University of Miami Health System can voluntarily enroll and consent to be contacted about clinical study opportunities for which they qualify through the Consent to Contact Registry managed by the University of Miami Clinical and Translational Science Institute. Potential participants were identified using the Clinical and Translational Science Institute Consent-to-Contact Registry through the generation authorized patient lists based on International Classification of Diseases (ICD-10) codes. Allo-SCT survivors were eligible to participate if they were (1) aged >18 years; (2) received an allo-SCT for myelodysplastic syndrome, acute myeloid leukemia, or chronic myeloid leukemia <36 months; and (3) were in remission >3 months. Survivors who (1) had a history of severe psychiatric illness (eg, psychosis, active suicidality, or inpatient treatment) in the past 12-months, (2) severe cognitive impairment (per the Short Portable Mental Status Questionnaire) [62], or (3) hearing impairment were excluded from the study. Patients who were referred by their physician or were identified by the consent-to-contact list were contacted by telephone by study staff to assess interest in participating and eligibility. Of the survivors (n=64) screened for eligibility, 34 were ineligible, 11 declined screening, and 7 did not consent to participate. A total of 12 survivors agreed to participate and provided informed consent; however, 1 participant was ill and unable to attend the focus group. The study team conducted 2 focus groups (focus group 1: n=6 and focus group 2: n=5) and 9 one-on-one usability and field-testing sessions.

#### Procedures

Before attending a focus group, participants completed a survey assessing sociodemographic (eg, age, gender, race, and income) and medical characteristics (eg, cancer type and time in remission). Focus groups were conducted through Zoom (Zoom Communications) and facilitated by 2 MT-BCs and study staff trained in qualitative methods. The aim of the focus groups was to understand the needs and burdens of allo-SCT patients and gather qualitative feedback to inform eMBMT content and platform design. Facilitators followed a semistructured interview guide including open-ended questions assessing: (1) challenges associated with receiving an allo-SCT (eg, uncertainty of the disease course, treatment-related side effects, and management of emotional and physical symptoms), (2) use of eHealth-based delivery for mindfulness, music therapy, and mindfulness-based music exercises, and (3) preferences for eMBMT length and detail of content. Facilitators also presented 2 modules from the eMBMT SmartManage platform and participants were asked about design and layout preferences (eg, theme, style, colors, and fonts), content detail and type (eg, text, images, and videos), and functionality features (eg, navigation, accessibility, and user interaction). Each focus group was facilitated by 2 experienced moderators (ie, “facilitators”) with training in qualitative research methods (SEF-P, BNE, TL, and MAK). To ensure balanced participation and that all voices were heard, the facilitators used strategies such as directly inviting quieter participants to share their thoughts, using prompts to encourage diverse perspectives, and managing dominant voices to maintain an inclusive environment [63,64]. Focus groups were used to capture a diverse range of perspectives and facilitate interactive discussions among participants, allowing for the exploration of shared experiences and unique viewpoints related to the intervention. This approach was chosen because participants could build on each other’s ideas, providing deeper insights into coping strategies and preferences. By leveraging the group dynamic, focus groups enabled the identification of common challenges and individual needs, ensuring the intervention was informed by both collective and personal experiences. Focus groups lasted approximately 3 hours and were audio recorded and detailed notes were taken to supplement the recordings.

Participants were contacted 1 week post–focus group participation to complete a usability and field-testing session. The usability assessment evaluated the ease of learning and identified platform design issues. Field testing evaluated both technical (eg, technical problems) and functional (eg, application use and integrating eMBMT into daily life) reliability. Sessions were conducted in-person or through Zoom and facilitated by study staff trained in usability and field testing of eHealth platforms. For usability testing, participants completed the 30-item USE questionnaire [65] which contains 4 subscales assessing usefulness (eg, “It helps me be more effective”), ease of use (eg, “It is easy to use”), ease of learning (eg, “I learned to use it quickly”), and satisfaction (eg, “I am satisfied with it”) on an 8-point Likert scale (1=strongly disagree to 8=strongly agree). For field testing, we used the “think aloud” method [66], which encourages participants to vocalize any thoughts or comments while performing or immediately after performing a task, such as reading or watching a video. Participants were asked to share their thoughts and reactions as they explored the eMBMT and platform. Finally, participants were prompted with questions addressing technical reliability (eg, technology problems) and functional reliability (eg, application use and integrating eMBMT into daily life). The usability and field-testing sessions lasted approximately 3 hours and were audio-recorded.

#### Data Analysis

The Usefulness, Satisfaction, and Ease of Use (USE) questionnaire [65] and sociodemographic and medical characteristics were analyzed with descriptive statistics. We applied rapid qualitative analysis (RQA) to analyze the focus groups and usability and field-testing sessions following Watkins’ guidelines [67]. Relative to traditional qualitative analysis methods, such as thematic analysis and grounded theory, which demand extensive time for tasks like multiple transcript readings and detailed coding, RQA is a more efficient and less time-demanding approach. RQA methods involve team members individually summarizing key points from qualitative data, and combining individual data by categorizing related key points to produce relevant themes [68]. RQA is a pragmatic, valid, and reliable method, which is crucial for guiding the refinement of digital interventions [68]. Study staff were trained on RQA, developed a coding matrix, individually coded the transcripts, and finally, met to complete a comprehensive final matrix summarizing data to facilitate the identification of themes. Themes were developed through iterative coding of the interview transcripts, with the team working to identify patterns and nuances in participant responses. While some themes aligned with the interview prompts, further analysis captured additional insights into coping mechanisms and patient preferences, which were critical for tailoring the intervention.

### Ethical Considerations

All procedures performed in studies involving human participants were in accordance with the ethical standards of the institutional and national research committee and with the 1964 Helsinki Declaration and its later amendments or comparable ethical standards. We complied with the following ethical considerations. First, we recruited and obtained informed consent from all participants before the focus groups and usability and field-testing sessions, as approved by the University of Miami’s Institutional Review Board (eProst 20230726). Participants were informed that their participation was voluntary, the focus groups and usability and field-testing sessions would be recorded, the collected information would be deidentified and securely stored, and they would receive compensation of US $50 for completing the focus group and US $50 for completing the usability and field-testing session, for a total of US $100. Second, we ensured that all transcript data were deidentified by replacing participant names with a unique identifier. All collected data are securely stored with access restricted to authorized researchers. Third, all researchers completed research compliance training through the Collaborative Institutional Training Initiative, ensuring adherence to best practices and ethical considerations in working with participants. This project is funded by the National Cancer Institute (1R61CA263335-01A1) and registered on ClinicalTrials.gov (NCT05968963).

## Results

### Participant Characteristics

Table 1 presents participant demographics and medical characteristics. Participants (N=11; mean age 43.6, SD 17.8; range 19‐76 years) were female (55%, n=6), White (91%, n=10), Hispanic (73%, n=8), with a history of acute myeloid leukemia (100%, n=11), and a mean time of 1.64 (SD 0.81) years post-SCT at the time of the focus groups.

**Table 1.** Demographics and medical characteristics of allogeneic stem cell transplantation (allo-SCT) survivors who participated in focus groups as part of the refinement of the eHealth-based mindfulness-based music therapy (eMBMT) intervention.

Demographics & medical characteristics	Total sample (N=11)	Focus group 1 (n=6)	Focus group 2 (n=5)
Age (years), mean (SD)	43.6 (17.8)	42.1 (15.9)	45.9 (18.4)
Gender, n (%)			
Female	6 (54.5)	4 (66.7)	2 (40)
Male	5 (45.5)	2 (33.3)	3 (60)
Race, n (%)			
White or Caucasian (including White Hispanic)	10 (91)	5 (83.3)	5 (100)
Black, African American, or Afro-Caribbean Black	1 (9)	1 (16.7)	0 (0)
Ethnicity, n (%)			
Hispanic	8 (72.7)	4 (66.7)	4 (80)
Non-Hispanic	3 (27.3)	2 (33.3)	1 (20)
Employment status, n (%)			
Full-time	5 (45.5)	3 (50)	2 (40)
Part-time	3 (27.3)	1 (16.7)	2 (40)
Unemployed with disability	2 (18.2)	2 (33.3)	0 (0)
Unemployed without disability	1 (9)	0 (0)	1 (20)
Education, n (%)			
High school or GED^a^	2 (18.2)	2 (33.3)	0 (0)
Some college	1 (9)	1 (16.7)	0 (0)
Graduated from a 2-year college	1 (9)	1 (16.7)	0 (0)
Graduated from a 4-year college	4 (36.5)	1(16.7)	3 (60)
Master’s degree	3 (27.3)	1 (16.7)	2 (40)
Cancer type, n (%)			
Acute myeloid leukemia	11 (100)	6 (100)	5 (100)
Treatment type, n (%)			
Allogeneic stem cell transplant	11(100)	6 (100)	5 (100)
Time since diagnosis (months), n	26	26.7	25.3

a General Education Development diploma.

### Focus Group Findings

Focus groups were conducted between April and May 2023. Qualitative analyses identified 4 themes.

#### The Initial Shock of a Cancer Diagnosis and Distress Over Unfavorable Outcomes

Most participants described the diagnosis of cancer as a profoundly shocking experience because they were diagnosed when they were feeling well and without any noticeable symptoms (“I didn’t know I was sick. I had regular blood work done April of 2021 and everything was normal. And then June 1st I had a lump like a lymph node in my neck that was a little enlarged, but I wasn’t feeling sick.”). In addition, some participants hesitated to seek care because they were worried about exposure to COVID-19, resulting in delayed diagnosis and treatment (“I had previous history of asthma. So, my concern was: I go to the hospital, I’m gonna die… it’s gonna hit people with respiratory problems, so I delayed it.”). Once diagnosed, they grappled with uncertainty, the fear of missing out on meaningful activities, and the pressure to make tough treatment decisions (eg, fertility-related choices). After not responding to the initial treatment and given the option to receive an allo-SCT, participants faced frustration and anxiety related to finding a stem cell match, coping with chemotherapy’s physical side effects, and age-related issues (eg, hearing and vision loss, reduced mobility).

#### Challenges Associated With Hospitalization, Loss of Normalcy, and Adjusting to Life After Treatment

During hospitalization, participants experienced psychological distress due to prolonged social isolation and lack of personal interactions with their family and friends (eg, “...So, my isolation turned into 60, 70, 90 days, so that made it even worse”). They had feelings of loneliness, anxiety, boredom, and frustration surrounding missing major life events such as graduation, prom activities, or their children’s events (eg, sport or school events). The COVID-19 pandemic restrictions negatively impacted the process of connecting patients to support services. Most participants reported a lack of access to support services during their hospital stay, which exacerbated their feeling of loneliness (eg, “I couldn’t see anyone because it was COVID. So, for 2, 3 months there was nothing. Nobody offered me anything”). Following the conditioning phase and the stem cell infusion, participants experienced multiple physical side effects including weight loss, hair loss, nausea, vomiting, diarrhea, mouth sores, and fatigue. These symptoms negatively impacted their daily activity levels during their hospital stay and after being discharged. They also indicated several psychosocial side effects including, fear of recurrence, anxiety, depression, feeling very different *(*eg, “I’m not even close to who I was before this happened to me”), as well as a substantial impact on body image and its associated stigma (eg, “I don’t want to be called the cancer kid at school”). They also noted that their families and friends were impacted by their disease (eg, “Another thing that I think it’s forgotten is about the children. Most of us, we have children, and they have seen us in the darkest moments of our lives… and you think that they wouldn’t remember, but they remember”).

#### Coping Strategies for Living With a Hematologic Cancer

Participants developed various coping strategies for managing their disease. Participants vocalized several perceived useful strategies, such as (1) seeking social support from family, friends, health care staff, and other patients; (2) denial (eg, “I don’t have cancer, I’m not going to admit it”); (3) spirituality and faith; (4) positive thinking and having an optimistic outlook; (5) goal setting; and (6) staying active and busy (eg, exercising, journaling, or watching television). In contrast, receiving adult coloring books and health communication materials was perceived as unhelpful or burdensome because they experienced changes in their ability to concentrate or memory lapses *(*“We don’t read the green pamphlet because it’s too much, too overwhelming, and you don’t have the time or the mind to think about that, you’re just literally surviving”). Support services were generally limited during their hospital stay due to COVID-19. However, when patients received supportive services, they valued the interaction with the therapist, provider, or both during those difficult times (eg, art therapy, music therapy, massage therapy, and physical therapy). In addition, although they mentioned that telehealth and videoconferencing were very convenient for maintaining continuity of care, they also experienced “screen fatigue.” Participants also noted the lack of follow-up after being discharged and that they had to request support services rather than being automatically referred to them. A participant mentioned that “there’s a waiting period of a year to see a sexologist in the gynecology department.”

#### Usefulness of Mindfulness and Music Therapy to Cope With Cancer

Participants perceived mindfulness as useful for staying positive, strengthening their spirituality, and coping with uncertainty. Although participants were open to practicing mindfulness through online means, they preferred in-person sessions and the ability to share their experiences with other patients. Participants perceived music therapy as helpful to change their mood, and bring joy, confidence, and faith. In contrast, a participant described herself as “not a music person” and that she “could go in life without music and would be fine.” Most participants were open to participating in online music therapy sessions; however, they underscored the need to account for patients’ physical and psychological symptoms, which could potentially impact their participation.

### Feedback on MBMT Prototype

Most participants agreed with the proposed number of sessions (n=8) and duration of the intervention (approximately 60 minutes/session). However, participants recommended implementing more sessions pretransplant to better help them be emotionally and psychologically prepared for the allo-SCT. They also suggested more sessions postdischarge to help them reintegrate into their day-to-day activities. Participants agreed with providing blood samples, but also suggested streamlining the process and obtaining samples along with already scheduled blood work. Participants described the eMBMT prototype within the SmartManage platform as “easy to read,” “easy to navigate,” “very straightforward,” and “very appealing.” Participants endorsed the order in which the mindfulness attitudes were presented (eg, “there is definitely a pattern, I think…There’s sort of a journey to it”). They believed that the in-person interaction with the music therapist would be very helpful. Participants recommendations included (1) incorporating diverse music genres(eg, “disco when you need to move” or “hip hop when you need to walk”), (2) allowing music selection based on patient’s mood, (3) recording audio or video clips to reduce the amount of text, (4) adding a sleep sounds feature, (5) reflecting the patient population within the content (eg, images specific allo-SCT concerns), (6) involving caregivers, (7) exploring a partnership with meditation and mindfulness apps, and (8) providing the necessary tools (eg, iPads or headphones).

### Usability and Field-Testing Session Findings

#### Overview

Usability and field-testing sessions were completed between June and July 2023. The 2 participants who completed the focus groups were unable to participate in usability and field-testing sessions. Participants (n=9) agreed that the eMBMT platform was useful (mean 6.47 SD 0.29), easy to use (mean 6.92, SD 0.60) and learn (mean 7.39, SD 0.75), and were satisfied with the platform (mean 6.16, SD 0.82).

Usability and field-testing sessions were completed between June and July 2023. Qualitative analysis identified 2 themes.

#### Participants’ Opinions About the MBMT Intervention

Overall, participants considered the program “helpful,” “easy to read,” and easy to navigate. They indicated that if the program had been provided during their hospitalization, they would have used it. Participants also liked the “concept” of the program (eg, holistic approach, accessibility, mindfulness meditation, and music to calm and distract from treatment). Regarding the aesthetics, participants perceived that the program was pleasant and appreciated personalized features (eg, their name on the front page). They also liked the colors, text size, layout, and images; although, 2 participants noted that the pictures did not represent the patient population accurately (eg, no photos of cancer patients without hair or racially diverse). Furthermore, some participants mentioned that the amount of text was overwhelming, section titles were confusing because they were very similar, and the platform lacked navigation guidance. They also mentioned that the exercises were repetitive with a lack of music options and that they could not have done some of the proposed activities during their hospital stay because of the physical symptoms (eg, mouth sores or extreme fatigue). Participants also experienced a few technical issues, such as some of the features not working (ie, audio), poor audio quality (ie, “white noise”), and slow internet connection.

#### Suggestions for Improving the eMBMT Intervention

Participants provided specific suggestions related to the content, including (1) reducing the amount of text by recording video and audio clips, (2) stating a specific goal at the beginning of each session (eg, this session will help you reduce your anxiety), (3) adding more relatable content (eg, “missing being outside,” “you may be feeling fatigued today. Here is what you can do”) and more practical knowledge (eg, “How will this help me?” and “Is there any section that helps you control pain by breathing more?”) rather than definitions (eg, “what is mindfulness?”), (4) connecting the mindfulness attitudes to patients’ experiences, (5) adding more guidance throughout the modules, (6) embedding YouTube videos with an introduction slide rather than links, (7) adding different music genres and the ability to choose, and (8) including more representative images. Participants also suggested new features such as (1) implementing a checkmark to indicate sections that have been reviewed, (2) allowing them to interact with other participants (eg, enabling a chat feature and support groups), (3) a journaling or writing section and a feedback box, (4) reducing the amount of clicking and scrolling, and (5) creating a Spotify playlist with the music from the program.

## Discussion

### Principal Findings

#### Overview

This study refined and finalized, using focus groups and environmental and field testing, an eMBMT intervention aimed at reducing symptom burden and improving HRQoL in patients undergoing allo-SCT. The focus group findings of this study underscore the significant emotional and physical challenges faced by allo-SCT patients, including the distress of diagnosis, hospitalization, and posttransplant recovery. These challenges align with previous research indicating that allo-SCT patients frequently experience psychological distress, including anxiety, depression, and feelings of isolation, which can negatively impact recovery and long-term survival [5,6,9]. Focus group findings also highlight that allo-SCT survivors had an appreciation for the potential positive impacts of the mindfulness and music therapy intervention components. In addition, the iterative refinement process, informed by usability and field-testing feedback, led to key modifications including enhanced content representativeness, reduced text with audio options, personalized music selection, and adjustments to session timing to better support patients pre- and posttransplant.

The development and refinement of the novel eMBMT intervention represents a significant step towards providing integrative interventions addressing HRQoL, symptom burden, disease activity, and psychosocial and physiological adaptation of adults undergoing allo-SCT. Grounded in theory and well-established therapeutic techniques (ie, MT, MM, and MBSR), the eMBMT intervention provides a structured delivery of in-person and eHealth sessions facilitated by an MT-BC, and is designed to be accessible and user-friendly. An eHealth intervention may be especially well suited for this population as shifting treatment phases and unexpected periods of isolation make it difficult to coordinate in-person support services.

The iterative process of refining the eMBMT content through focus groups and usability and field-testing sessions was crucial for its refinement. Engaging allo-SCT survivors in the development process allowed for the collection of valuable data that directly informed the content, design, and functionality of the intervention. Overall, participants reported positive experiences with the platform and described the content as useful and most participants expressed that they would have participated in such an intervention had it been available at the time of their treatment.

The qualitative analysis identified key themes that highlight the complex emotional and physical challenges faced by allo-SCT patients. These include the initial shock of a cancer diagnosis, the distress associated with hospitalization and treatment, and the ongoing struggle to regain normalcy posttransplant. Participants also shared coping strategies, underscoring the importance of social support, positive thinking, and maintaining an active lifestyle. The perceived usefulness of mindfulness and music therapy further validates the inclusion of these components in the eMBMT intervention.

The use of rapid qualitative analysis enabled the timely synthesis of participant feedback, which was essential for adapting the intervention to the unique needs of patients undergoing allo-SCT. Rapid qualitative analysis has been increasingly recognized as a valid and efficient method for evaluating eHealth interventions and has been successfully used in previous studies to inform digital health tools [67,68]. While a traditional qualitative approach might have provided additional granularity, the chosen method ensured that the findings were actionable within the project’s timeline. We acknowledge that rapid qualitative analysis may result in themes that closely reflect the structure of the interview prompts. To address this, the research team used iterative coding and thematic refinement to ensure the themes captured meaningful nuances and extended beyond the initial prompts, thereby providing deeper insights into participants’ experiences and needs.

The feedback from usability and field-testing sessions was overwhelmingly positive, with participants rating the platform highly in terms of usefulness, ease of use, and satisfaction. However, several areas for improvement were identified, which were invaluable for further refining the intervention to better meet the needs of patients. Below we discuss the key areas of improvement and subsequent modifications made to the eMBMT content and intervention.

#### Enhance the Representativeness of the Content

More representative images of the patient population were included within the eMBMT platform content (eg, an image of a cancer patient in hospital robe listening to music, patients without hair, more racially and ethnically diverse).

#### Reduce the Amount of Text and Reading

We reduced the amount of text and provided an option to listen to content. All intervention content was professionally audio-recorded and integrated into the respective section of the platform.

#### Provide Headphones

All participants will now receive a pair of personal headphones for ease of listening to content.

#### Add More Guidance Throughout the Modules

Additional instructions guiding participants through the modules were added.

#### Modify the eMBMT Session Delivery Timeline

The eMBMT session delivery timeline was modified such that more sessions are provided pretransplant to aid with the emotional and psychological preparation for the allo-SCT. In addition, more sessions are provided postdischarge to help with reintegration into their day-to-day activities. Table 2 presents the sessions, content topics, and the delivery timeline.

**Table 2.** Overview of the 8-session eHealth-based mindfulness-based music therapy (eMBMT) intervention for allogeneic stem cell transplantation (allo-SCT) patients, detailing session content, mindfulness principles, and time relative to the transplant.

Session	SCT^a^ treatment phase	Mindfulness attitude	MT^b^ component	Attitude carry over after eMBMT
1	Admission	MBMT^c^ Orientation	Breathing to music	Focus on the present
2	Conditioning	Nonjudging	Music listening and journaling	Unpleasant and pleasant experiences and writing
3	Early post transplant	Patience	Focused music and environmental sound listening, simple body movements to music	Awareness and experience of patience
4	Early post transplant	Letting go	Music-assisted relaxation and scripts	Discussion and journaling of letting go
5	Engraftment to discharge	Acceptance	Music-assisted relaxation and body scan	Nonjudgmental awareness and acceptance of physical sensations, thoughts, and feelings
6	Postdischarge	Trust	Vocal improvisation	Trusting oneself
7	Postdischarge	Beginner’s mind	Novel instrument sounds	Discussion and journaling of beginner’s mind
8	Postdischarge	Nonstriving	Music improvisation	Focus on the process, letting go of preconceived goals with what “should be”

a SCT: stem cell transplantation.

b MT: music therapy.

c MBMT: mindfulness-based music therapy.

#### Include Different Genres of Music and Preferred Music

Participants communicated the desire for a more varied genre of music and the ability to choose their preferred music. Thus, more diverse samples of music were included as well as allowing participants to choose their preferred music to complete eMBMT activities.

#### Provide a Journaling Section

Modules now provide sections within each module where participants can journal, write their thoughts, or take notes.

#### Streamline Blood Sample Collection

The blood sample collection was streamlined such that at baseline, blood is collected only after the patient has received their port and when the patient is scheduled for a follow-up appointment post-discharge.

#### Address Technical Issues

All technical issues (eg, audio was not functioning properly) were resolved. Furthermore, audio that was deemed of poor quality (ie, “white noise”) was professionally re-recorded.

Feedback from the future pilot randomized controlled trial will shed light on potential further modifications to the eMBMT content and intervention as well as provide examples of how participants successfully implement mindfulness attitudes in their own lives throughout their allo-SCT treatment.

### Limitations

This study has several limitations. First, the sample size was small and primarily consisted White and Hispanic individuals, which may limit the generalizability of the findings to more diverse populations undergoing allo-SCT. Second, while the focus groups and usability and field testing provided valuable qualitative insights, the results may be influenced by selection bias, as participants who opted into the study may have been more motivated or receptive to mindfulness and music therapy interventions. Third, the rapid qualitative analysis approach, while efficient, may have limited the depth of thematic exploration compared to more traditional qualitative methodologies. Finally, while usability testing identified technical issues and design preferences, future iterations should consider additional real-world implementation challenges, including potential barriers to patient engagement with digital health interventions.

### Conclusion

The development and refinement of the eMBMT intervention through focus groups and usability and field testing has resulted in an eHealth intervention for use with allo-SCT patients. The study findings underscore the importance of patient involvement in intervention design, ensuring that content is both relevant and engaging. As digital interventions become increasingly prevalent in health care, eMBMT can serve as a model for leveraging technology to deliver complementary, evidence-based psychosocial support. Future research should explore its long-term impact, scalability, and integration into standard transplant care to optimize patient outcomes and reduce health care burdens. A future pilot randomized controlled trial will evaluate the feasibility, acceptability, and preliminary efficacy of the eMBMT on health-related quality of life, symptom burden, disease activity, and psychosocial and physiological adaptation [69].

## Abbreviations

allo-SCT: allogeneic stem cell transplantation
eMBMT: eHealth-based mindfulness-based music therapy
GVHD: graft-versus-host disease
HIPAA: Health Insurance Portability and Accountability Act
HRQoL: health-related quality of life
*ICD*: *International Classification of Diseases*
MBMT: mindfulness-based music therapy
MBSR: mindfulness-based stress reduction
MM: mindful meditation
MT: music therapy
MT-BC: board-certified music therapist
RQA: rapid qualitative analysis
USE: Usefulness, Satisfaction, and Ease of Use
